# Estrogen Receptor-α36 Is Involved in Pterostilbene-Induced Apoptosis and Anti-Proliferation in *In Vitro* and *In Vivo* Breast Cancer

**DOI:** 10.1371/journal.pone.0104459

**Published:** 2014-08-15

**Authors:** Chi Pan, Yiwang Hu, Jun Li, Zhaoyi Wang, Jianjin Huang, Suzhan Zhang, Ling Ding

**Affiliations:** 1 Cancer Institute, Second Affiliated Hospital, Medicine School of Zhejiang University, Hangzhou, Zhejiang, China; 2 Department of Surgical Oncology, First Affiliated Hospital, Wenzhou Medical University, Wenzhou, Zhejiang, China; 3 Department of Surgical Oncology, Second Affiliated Hospital, Medicine School of Zhejiang University, Hangzhou, Zhejiang, China; 4 Department of Medical Microbiology & Immunology, Medical School of Creighton University, Omaha, Nebraska, United States of America; 5 Department of Medical Oncology, Second Affiliated Hospital, Medicine School of Zhejiang University, Hangzhou, Zhejiang, China; H.Lee Moffitt Cancer Center & Research Institute, United States of America

## Abstract

Pterostilbene (*trans*-3,5-dimethoxy-4′-hudroxystilbene) is an antioxidant primarily found in blueberries. It also inhibits breast cancer regardless of conventional estrogen receptor (ER-α66) status by inducing both caspase-dependent and caspase-independent apoptosis. However, the pterostilbene-induced apoptosis rate in ER-α66-negative breast cancer cells is much higher than that in ER-α66-positive breast cancer cells. ER-α36, a variant of ER-α66, is widely expressed in ER-α66-negative breast cancer, and its high expression mediates the resistance of ER-α66-positive breast cancer patients to tamoxifen therapy. The aim of the present study is to determine the relationship between the antiproliferation activity of pterostilbene and ER-α36 expression in breast cancer cells. Methyl-thiazolyl-tetrazolium (MTT) assay, apoptosis analysis, and an orthotropic xenograft mouse model were used to examine the effects of pterostilbene on breast cancer cells. The expressions of ER-α36 and caspase 3, the activation of ERK and Akt were also studied through RT-PCR, western blot analysis, and immunohistochemical (IHC) staining. ER-α36 knockdown was found to desensitize ER-α66-negative breast cancer cells to pterostilbene treatment both *in vitro* and *in vivo*, and high ER-α36 expression promotes pterostilbene-induced apoptosis in breast cancer cells. Western blot analysis data indicate that MAPK/ERK and PI3K/Akt signaling in breast cancer cells with high ER-α36 expression are mediated by ER-α36, and are inhibited by pterostilbene. These results suggest that ER-α36 is a therapeutic target in ER-α36-positive breast cancer, and pterostilbene is an inhibitor that targets ER-α36 in the personalized therapy against ER-α36-positive breast cancer.

## Introduction

Breast cancer is the most common malignant tumor in women, and its incidence in the world is persistently rising [1]. It is a hormone-related systemic disease, and endocrine therapy effectively blocks its estrogen receptor (ER-α66, the classic estrogen receptor) pathway to inhibit tumor progression. ER-α66 expression is an important indicator of breast cancer, and previous studies show that patients with ER-α66-negative tumors have shorter disease-free intervals and worse overall survival than patients with ER-α66-positive tumors [2], [3]. Tamoxifen (TAM, the selective estrogen receptor modulator) is the most effective drug commonly used for the endocrine therapy of ER-α66-positive breast cancer patients [4]. Patients with ER-negative-tumors are therefore not supposed to respond to TAM therapy. However, clinical studies show that most ER-positive tumors eventually resist TAM therapy despite initial responsiveness to TAM [5], [6]. Finding new therapeutic strategies is an urgent topic in breast cancer research nowadays.

Pterostilbene (*trans*-3,5-dimethoxy-4′-hydroxystilbene; Pter) is an anti-oxidant predominantly found in blueberries, some types of grapes, and tree wood [7], [8]. It is a natural dimethylated analog of the well-studied resveratrol (3,5,4′-trihydroxystilbene), which is a naturally occurring polyphenol that shows pleiotropic health benefits, including anti-oxidant, anti-inflammatory, anti-aging, cardioprotective, and neuroprotective activities[9]. However, pterostilbene is significantly more bioavailable than resveratrol after being ingested [10], [11]. Pterostilbene has been shown to be an effective antioxidant in multiple cancer cell lines, which may facilitate its function as an anticarcinogenic agent. Pterostilbene inhibits breast cancer through mitochondrial depolarization and the induction of caspase-dependent [12], [13], [14] and caspase-independent apoptosis [15]. Phosphatidylinositol 3-kinase (PI3K)/serine/threonine kinase (Akt) activation, the vital pathway associated with accelerated cell proliferation, was found to be decreased in cancer cells after pterostilbene treatment [16], [17]. Chen *et al*. also reported that pterostilbene-treated lung cancer cells decreases extracellular regulated protein kinases 1/2 (ERK1/2) activation via the inhibition of EGFR-mediated pathways and apoptosis induction [17]. Preliminary studies also demonstrate that pterostilbene induces apoptosis and inhibits cell viability in both ER-α66-positive and ER-α66-negative breast cancer cell lines[18].

Wang *et al*. have recently identified and cloned a 36-kDa variant of ER-α66, named ER-α36, which is expressed in ER-negative tumor tissues and ER-negative breast cancer cell lines [19], [20], [21]. ER-α36 and ER-α66 are splicing variants from the same gene, but the previously unidentified promoter of ER-α36 is located in the first intron of ER-α66, indicating that ER-α36 expression is subject to a transcriptional regulation different from ER-α66 [22]. ER-α36 is also predominantly localized in both the plasma membrane and cytoplasm, and it may be associated with both genomic and non-genomic signaling networks [19], [23], [24]. It therefore is a compound of interest, and possibly is a new therapeutic target in breast cancer.

Alosi *et al*. showed that the pterostilbene-induced apoptosis in ER-α66-negative breast cancer cells MDA-MB-231 is more obvious than in ER-α66-positive breast cancer cells MCF-7 [14]. ER-α36-mediated mitogenic estrogen signaling in ER-negative breast cancer cells, such as MDA-MB-231, was previously found to lack ER-α66 expression, but highly express ER-α36 [25]. However, the exact mechanism of pterostilbene in ER-α66-negative breast cancer cell apoptosis is still under investigation.

In this study, ER-α36 is reported to play an important role in mediating the *in vivo* and *in vitro* pterostilbene-induced apoptosis of breast cancer cells. ER-α36 was also found to mediate reducing the activation of the Akt and Erk1/2 pathway using pterostilbene.

## Materials and methods

### Ethical statement

All of the animal experiments described in this study were approved by the Institutional Animal Care and USE Committee (IACUC) at Zhejiang University. All animals were maintained in accordance with the IACUC guideline.

### Chemicals and antibodies

Monoclonal anti-ER-α36 antibody was kindly provided by Professor Zhaoyi Wang (Department of Medical Microbiology & Immunology, Creighton University Medical School, Omaha, Nebraska, USA). Estrogen receptor α antibody, cysteinyl aspartate specific proteinase 3 (caspase 3) antibody, Akt antibody, phospho-Akt antibody, extracellular regulated protein kinases 1/2 (ERK1/2) antibody, anti-phosphor-ERK1/2 (Thr^202^/Tyr^204^) antibody, and anti-β-actin antibody were purchased from Cell Signaling Technology (Danvers, MA). Pterostilbene was obtained from Merck KGaA (Darmstadt, Germany). Geneticin (G418), insulin, and AnnexinV-FITC Apoptosis Detection Kit were obtained from Sigma-Aldrich (St. Louis, MO).

### Cell culture and reagents

The cell lines MDA-MB-231 with ER-α36 expression knocked down, MCF-7 transfected with high expression of ER-α36, MDA-MB-231, and MCF-7 transfected with control vectors were named as Mb231/Si36, MCF-7/ER36, Mb231, and MCF-7, respectively, in this article. These cells were stably established through the method described before [25], [26], and all were kindly provided by Professor Zhaoyi Wang, Creighton University. All parental and derivative cells were cultured at 37°C in a 5% CO_2_ atmosphere in a humidified incubator. Mb231/Si36 and Mb231 were maintained in L-15 media (Gibco) supplemented with 10% fetal calf serum and 250 µg/ml G418. MCF-7/ER36 and MCF-7 were maintained in DMEM (Gibco) supplemented with 10% fetal calf serum, 250 µg/ml G418, and 10 µg/ml insulin, following ATCC culture methods.

### RNA purification and RT-PCR

Total RNA was prepared using the “TRIzol” RNA purification reagent. The cDNA was synthesized through the reverse transcription of mRNA using oligo(dT) 20 primer and SuperScript III Reverse Transcriptase (Invitrogen). The RT-PCR analysis of ER-α36 and β-actin was performed using gene specific primers, described before as the following [25]. ER-α36: forward primer: 5′-CAAGTGGTTTCCTCGTGTCTAAAG-3′; reverse primer: 5′-TGTTGAGTGTTGGTTGCCAGG-3′; β-actin: forward primer: 5′-CCTGGCACCCAGCACAAT-3′; reverse primer: 5′-GCTGATCCACATCTGCTGGAA-3′. The PCR was performed using the PCR Master Mix kit (Beyotime, Nantong, China), according to the manufacturer protocol. PCR products were analyzed by electrophoresis in a 2.5% agarose gel, and were visualized by ethidium bromide staining under UV illumination. The relative mRNA expression was determined from the optical density (OD) ratio of the corresponding mRNA bands, determined using BandScan 5.0 software (Glyko inc., Upper Heyford, UK).

### Methyl-thiazolyl-tetrazolium (MTT) assay

Mb231/Si36, Mb231, MCF-7/ER36, and MCF-7 cells were seeded at a density of 3×10^3^/well in 96-well plates, and were incubated at 37°C overnight. The cells were then treated with various concentrations of pterostilbene for 72 h. Cell growth was measured by adding 20 µl of 5 mg/ml 3-(4,5-dimethylthiazol-2-yl)-2,5-diphenyltetrazolium bromide (MTT) to each well, and the plates were incubated at 37°C for 4 h. The supernatant was removed afterwards, and the formazan crystals were dissolved in 200 µl dimethylsulfoxide (DMSO) for 15 min. Absorbance was then measured at 570 nm wavelength using a multiwell spectrophotometer (Bio-Rad). Cell viability is expressed as the percentage of surviving pterostilbene-treated cells *vs*. control cells (whose viability was considered 100%). The experiments were independently performed in triplicate.

### Apoptosis analysis

Apoptosis was detected using an AnnexinV-FITC Apoptosis Detection Kit (Sigma-Aldrich, St. Louis, MO). Mb231/Si36, Mb231, MCF-7/ER36, and MCF-7 cells were treated with 30 µM pterostilbene for 72 h before apoptosis analysis. Cells were harvested by trypsinization, washed twice with PBS, and incubated in 500 µL of binding buffer and 10 µL of Annexin V–FITC at room temperature for 30 min. Subsequently, 5 µL of PI was added, and the cells were incubated for 5 min. Apoptosis data were collected using flow cytometry (BD FACSCanto II, BD Biosciences, San Jose, CA, USA), and were analyzed using the CellQuest software (Becton Dickinson, Franklin Lakes, NJ, USA). The experiments were independently performed three times.

### Western blot analysis

Whole cell extracts were obtained by treating cells with RIPA buffer (50 nM Tris pH 8.0, 150 mM NaCl, 1% Triton X-100, 0.5% Sodium deoxycholate, 0.1% SDS, 2 mM EDTA, and 5% Glycerol) containing a protease and phosphatase inhibitors cocktail (Sigma-Aldrich, St. Louis, MO, USA) for 30 min on ice. Appropriate protein extracts of cell lysates were fractionated through SDS-PAGE and were electro-transferred to polyvinylidene difluoride (PVDF) membranes (Millipore, Billerica, MA, USA). The membranes were probed with various primary antibodies and HRP-conjugated secondary antibodies, and were visualized using enhanced chemiluminescence (ECL) detection reagents (Millipore, Billerica, MA, USA). The molecular weights of the immunoreactive proteins were estimated based on the PageRuler Prestained Protein ladder (MBI Fermentas, USA). Experiments were repeated at least three times.

### Orthotopic xenograft assay

Five to six weeks old female nude mice (01B74-Athymic NCr-nu/nu) were ordered from the Experimental Animal Center of Zhejiang Chinese Medical University, and were housed in cages with wood chip beddings in a temperature-controlled room (68°F to 72°F) with a 12-h light-dark cycle and 45% to 55% relative humidity, and were permitted free access to diet and drinking water. Mice were injected with 1.6×10^6^ Mb231/Si36 and Mb231 cells suspended in 100 µl of L-15 medium containing 50% Matrigel (BD Bioscience) into one their left and right breast pads, respectively. The mice were randomly assigned to experimental and control groups (n = 3) when the tumors reached the size of ∼50 mm^3^. The mice were fasted overnight, and were administered with 56 mg/kg pterostilbene or physiological saline (vehicle) by oral gavage once every four days for 3 weeks, as the methods described before [27]. Tumors were measured using calipers once every two days, and the tumor volumes were calculated (Volume (mm^3^)  =  π× length × width^2^/6). At the end of study, tumors were harvested, fixed, and embedded in paraffin. Tumor sections were subjected to standard H&E staining and immunohistochemical (IHC) staining using anti- ER-α36 antibody. The experiment was conducted with replication.

### Immunohistochenical analysis

Immunohistochemical (IHC) analysis of ER-α36 expression was performed using 5 µm-thick paraffin-embedded tumor sections. The tissue sections were dewaxed and heated for 20 min with EDTA (pH 9.0) for antigen retrieval, endogenous peroxidase activity was quenched with 3% H_2_O_2_ for 10 min at 37°C, and the slides were incubated with normal goat blood serum to block nonspecific binding sites. The slides were rinsed in phosphate-buffered saline, and were incubated for 2 h at 37°C with anti-ER-α36 antibody at 1∶100 dilution. The slides were then washed with PBS and were stained with horseradish peroxidase (HRP)-conjugated rabbit anti-mouse antibodies (Santa Cruz Biotechnology, Santa Cruz, CA) for 1 h. The slides were exposed to DAB after washing with PBS. The slides were counterstained using hematoxylin, and were coverslipped with neutrogum.

### Statistical analysis

Data are expressed as mean ± standard error (SE). Two-tailed Student's t-tests were used for analyzing statistical differences between two groups using IBM SPSS software (SPSS, Chicago, IL, USA), and the significance was set at p<0.05. Graphs were generated using GraphPad InStat software program (GraphPad Software, La Jolla, CA, USA).

## Results

### Breast cancer cells with high ER-α36 expression were sensitive to *in vitro* pterostilbene treatment

**Figure 1:**
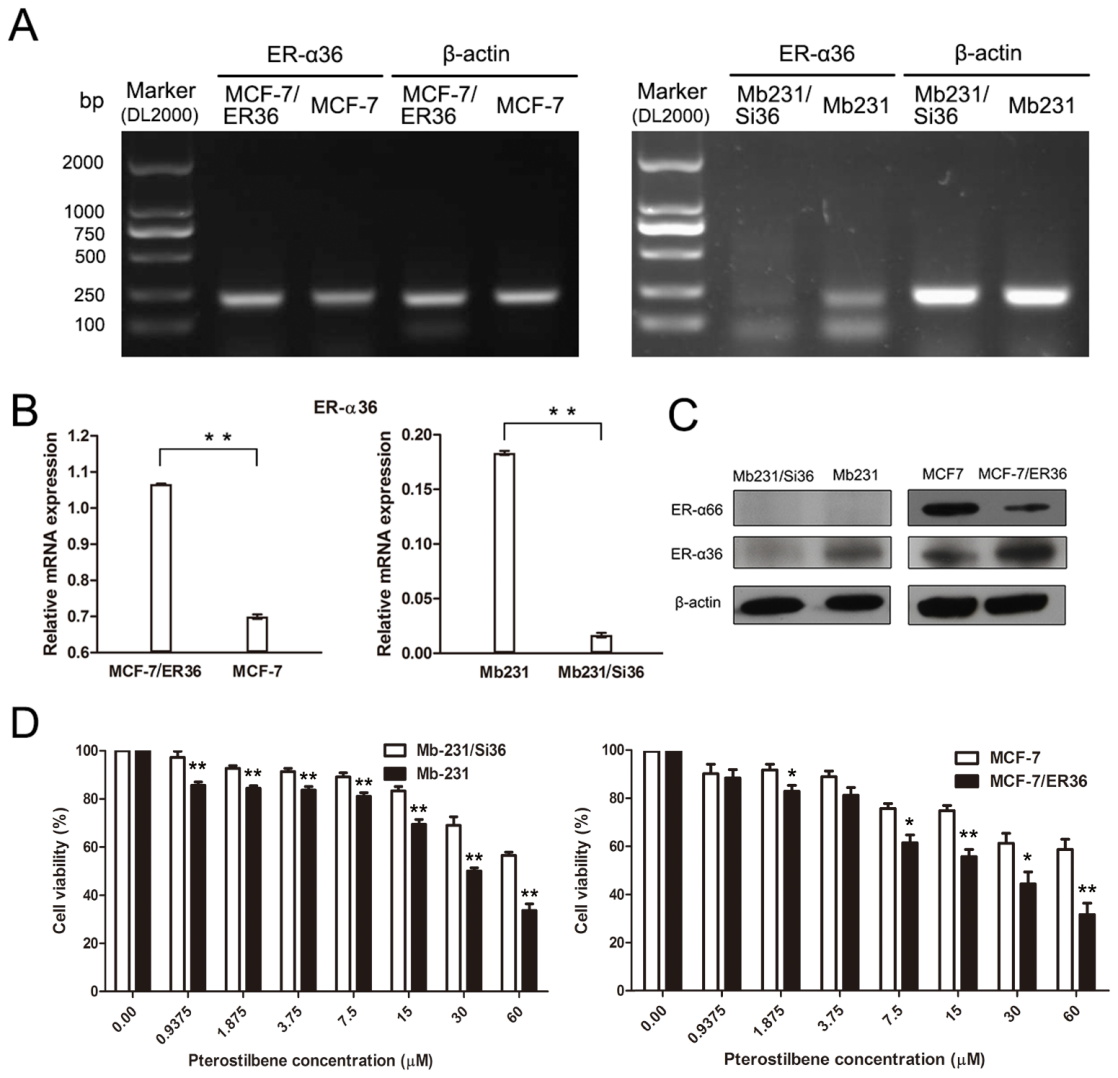
High ER-α36 expression sensitizes breast cancer cells to pterostilbene treatment. **a**. ER-α36 mRNA levels were measured in Mb231, Mb231/Si36, MCF-7, and MCF-7/ER36 cells using RT-PCR. **b**. The relative mRNA expressions are presented as the ratio of the OD of the corresponding mRNA bands compared to the OD of the β-actin bands. **c**. Western blots of ER-α36 and ER-α66 proteins in Mb231, Mb231/Si36, MCF-7, and MCF-7/ER36 cells using β-actin levels as the loading control. **d**. MTT assays measuring the cell proliferation of the two paired cells after control and pterostilbene treatments for 72 h. The relative cell numbers were normalized to those of their respective pterostilbene untreated controls (set as 100%). (Bars, SE. *p<0.05, **p<0.01).

RT-PCR was performed to determine ER-α36 gene expression. The ER-α66 and ER-α36 protein expressions in these breast cancer cells were analyzed through Western blotting. The PCR amplicon obtained was the same size as that described before [25]. The ER-α36 gene expression in Mb231/Si36 with ER-α36 knocked down was dramatically decreased compared to parental Mb231 cells. MCF-7/ER36 overexpressed ER-α36 compared to MCF-7 cells. β-actin gene expression was used as the internal control (Fig. 1A). The relative ER-α36 mRNA expressions were determined using the ratio of the OD of mRNA bands compared and the OD of the corresponding β-actin bands (Fig. 1B). Data from RT-PCR are consistent with the protein levels determined through western blot analysis (Fig. 1C). The ER-α66 protein expressions in Mb231 and Mb231/Si36 were undetectable, whereas it decreased in MCF-7/ER36 cells, compared with that in parental MCF-7 cells (Fig. 1C).

Whether the sensitivity of breast cancer cells to pterostilbene dependd on ER-α36 expression was determined. MTT assay was performed and Mb231/Si36 with negative ER-α36 expression were found to exhibit dramatically decreased sensitivity to pterostilbene compared to the parental Mb231 cells. MCF-7/ER36 cells with ER-α36 overexpression were also more sensitive to pterostilbene than MCF-7 cells (Fig. 1D).

These data indicate that higher ER-α36 expression increases the sensitivity of breast cancer cells to pterostilbene.

### High ER-α36 expression promotes pterostilbene-induced apoptosis in breast cancer cells

**Figure 2:**
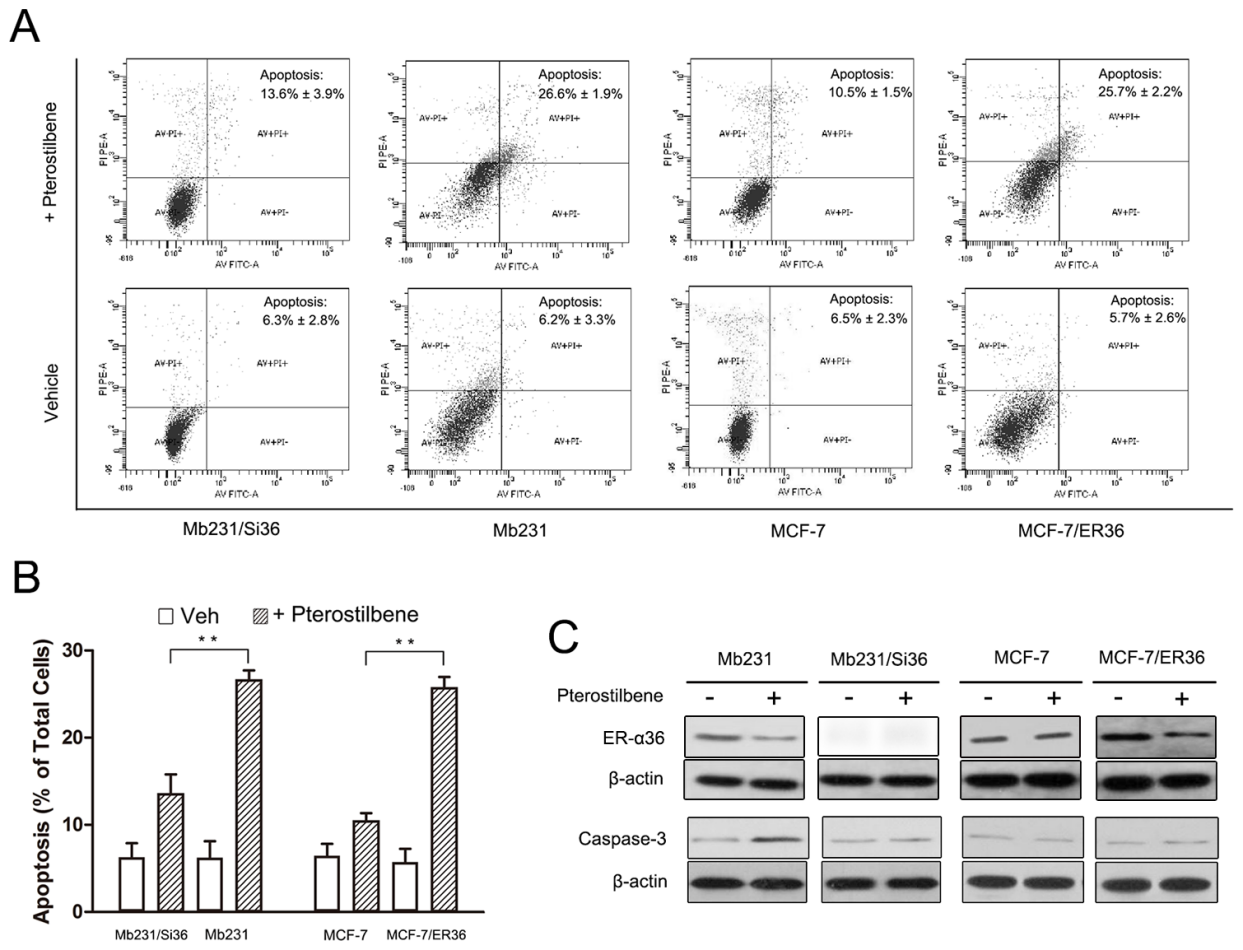
High ER-α36 expression enhances pterostilbene-induced apoptosis in breast cancer cells. **a**. Apoptosis induction in Mb231, Mb231/Si36, MCF-7, and MCF-7/ER36 cells treated with 30 µM pterostilbene for 72 h was analyzed through flow cytometry (X-Axis: Annexin V; Y-Axis: Propidium iodide, PI). The apoptosis rates are presented as both early and late apoptotic cells compared to total cells. **b**. Statistical analysis of FACS in (**a**). **c**. Caspase-3 expression in the two paired cells after control and 30 µM pterostilbene treatment for 72 h. (Means ± SE, **p<0.01).

Pterostilbene induced apoptosis in conventional ER (ER-α66)-positive breast cancer cells MCF-7 and ER-negative breast cancer cells Mb231 [12], [14]. To investigate whether ER-α36 affects pterostilbene-induced apoptosis, apoptosis in Mb231, Mb231/Si36, MCF-7, and MCF-7/ER36 cells were analyzed after treatment with 30 µM pterostilbene for 72 h. Apoptosis was confirmed with Annexin V-FITC/PI dual staining using FACSCanto II (BD Biosciences). Both early and late apoptotic cells were found to be significantly increased after pterostilbene treatment (Figs. 2A and B). Julie et *al*. demonstrated that pterostilbene increases caspase-dependent apoptosis, and its effect on Mb231 cells is more obvious than on MCF-7 cells [14]. The apoptotic percentage of Mb231 cells was consistently higher than that of MCF-7 cells (26.6±1.9% versus 10.5±1.5%) (Fig. 2A) in this study. Western blot analysis also revealed that pterostilbene inhibits ER-α36 expression in ER-α36-positive Mb231 cells and ER-α36-overexpressing MCF-7/ER36 cells and increases caspase-3 expression in Mb231 cells, but not in MCF-7 cells (Fig. 2C). The apoptotic percentage of Mb231/Si36 with ER-α36 knocked down induced by pterostilbene was decreased compared to parental Mb231 cells (26.6±1.9% versus 13.6±3.9%, *p* = 0.006) (Figs. 2A and B). Pterostilbene did not effectively induce caspase-3 expression in Mb231/Si36 cells (Fig. 2C). The apoptosis induced by pterostilbene was similarly promoted in MCF-7/ER36 cells with higher ER-α36 expression compared to the parental MCF-7 cells (25.7±2.2% versus 10.5±1.5%, *p* = 0.006) (Figs. 2A and B). However, the caspase-3 expression was not significantly increased in both MCF-7 and MCF-7/ER36 cells after pterostilbene treatment (Fig. 2C). These data indicate that pterostilbene-induced apoptosis depends on high ER-α36 expression in breast cancer cells.

### Pterostilbene deactivates ER-α36-mediated MAPK/ERK and PI3K/Akt signaling in breast cancer cells

**Figure 3:**
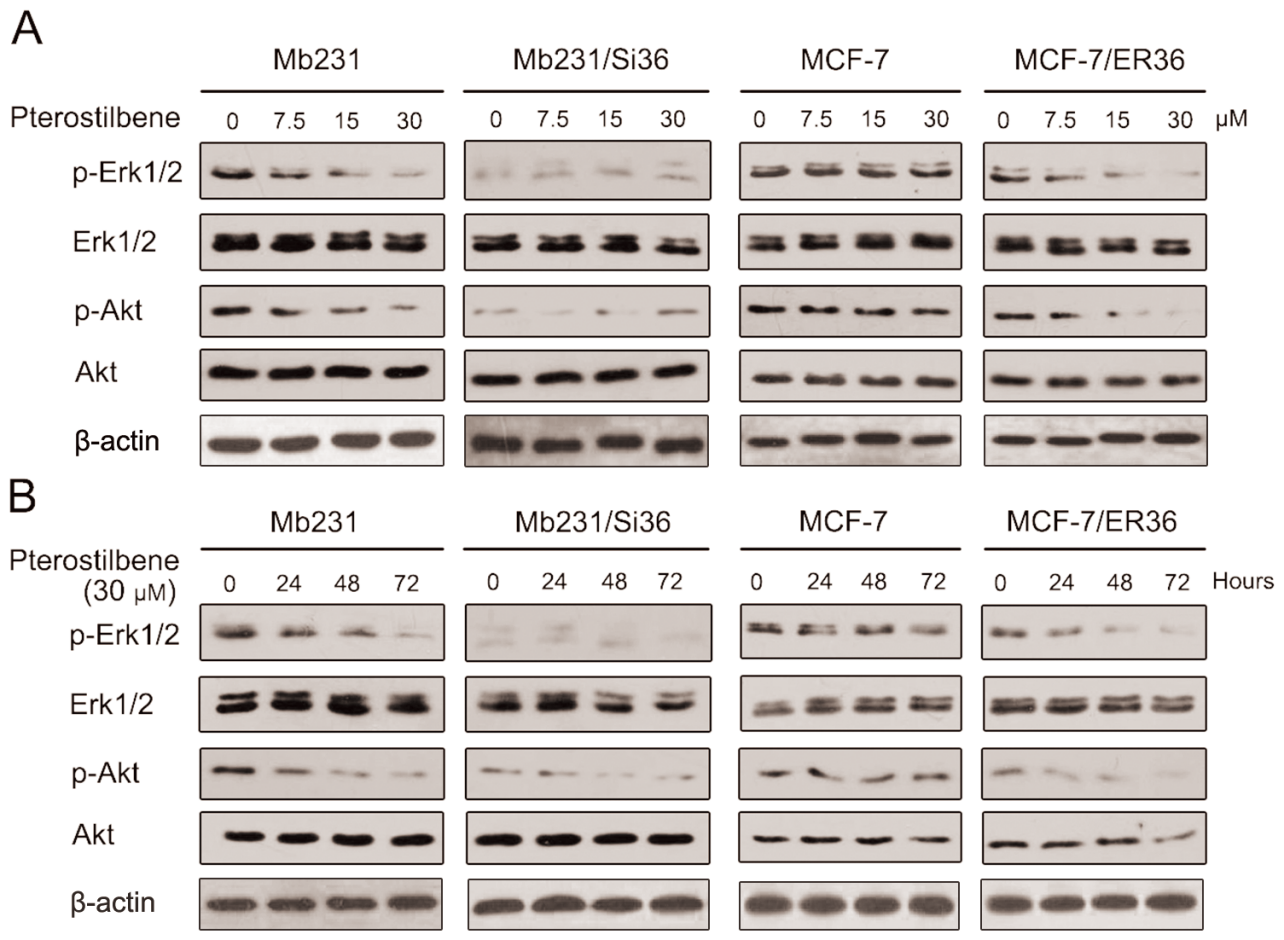
Pterostilbene inhibits the ERK1/2 and Akt activation via ER-α36 in breast cancer cells. **a**. Mb231, Mb231/Si36, MCF-7, and MCF-7/ER36 cells were treated with the indicated concentrations of pterostilbene for 72 h. **b** Mb231, Mb231/Si36, MCF-7, and MCF-7/ER36 cells were treated with 30 µM pterostilbene for the indicated hours. Changes in p-ERK1/2 and p-Akt protein levels were analyzed through western blotting. The total ERK1/2 and Akt proteins were used as loading controls.

Previous studies demonstrate that pterostilbene decreases ERK1/2 and Akt activation in cancer cells [16], [17]. The expression of ER-α36 is also related to MAPK/ERK and PI3K/Akt signaling in breast cancer [19], [25], [28], [29]. These findings suggest that ERK1/2 and Akt activation is initiated by non-genomic signaling via ER-α36. The MAPK/ERK and PI3K/Akt phosphorylation levels in pterostilbene-treated Mb231, Mb231/Si36, MCF-7 and MCF-7/ER36 cells were examined in this study to investigate the role of ER-α36 in the regulation of the reduced activation of ERK1/2 and Akt after pterostilbene treatment. The cells weret treated with different concentrations pterostilbene (7.5 µM, 15 µM, and 30 µM) for 72 h before Western blot analysis. ERK1/2 and Akt phosphorylation was then found to decrease in a dose-depended manner in ER-α36-positive Mb231 and MCF-7/ER36 with ER-α36 overexperssion, but not in MCF-7 cells (Fig. 3A). The cells were treated with 30 µM pterostilbene for 24 h, 48 h, and 72 h to determine if the inhibition of ERK1/2 and Akt activation is in a time-dependent manner. Phosphor-ERK1/2 and phosphor-Akt expressions were found to be inhibited in a time-depended manner in Mb231 and MCF-7/ER36 cells, but not in MCF-7 cells (Fig. 3B). The p-ERK1/2 and p-Akt expressions in Mb231/Si36 were nearly undetected with or without pterostilbene treatment. These results indicate that ER-α36 mediates pterostilbene to inhibit MAPK/ERK and PI3K/Akt phosphorylation in breast cancer cells, and ER-α36 knockdown totally deactivates ERK1/2 and Akt phosphorylation in Mb231/Si36 cells.

### Silencing ER-α36 reduces the sensitivity of the xenograft tumors of ER-α36-positive breast cancer to pterostilbene *in vivo*

**Figure 4:**
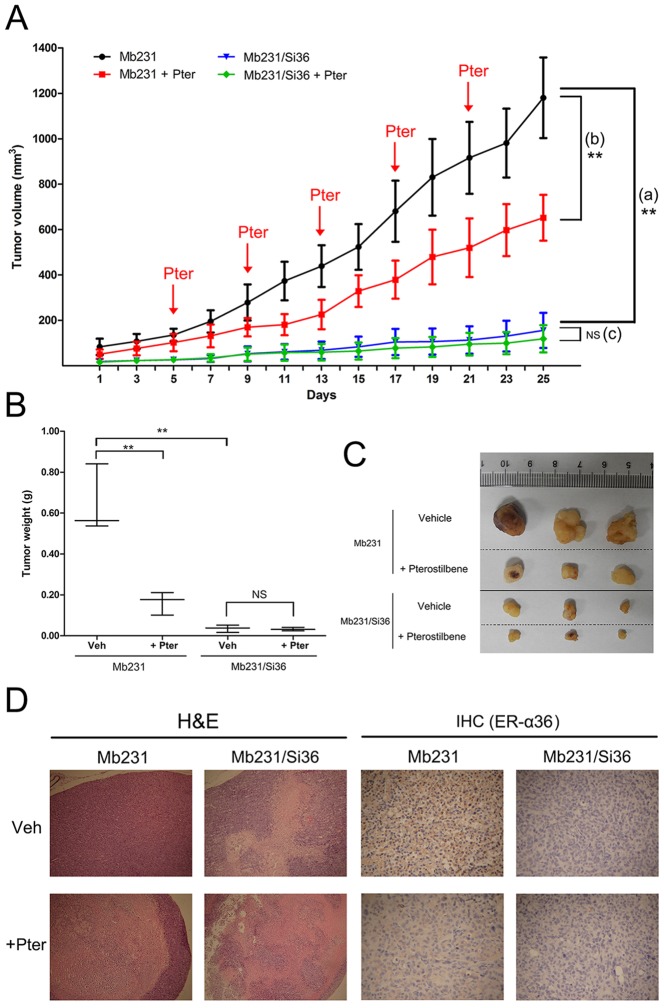
ER-α36 knockdown reduces the sensitivity of the xenograft tumors of ER-α36-positive breast cancer to pterostilbene. Mb231/Si36 and Mb231 cells were injected into the left and right breast pad, respectively of 5 to 6 weeks old nude mice. When the tumors reached about 50 mm^3^ size, the mice were randomly assigned into two groups (n = 3): vehicle control (Veh) and pterostilbene treatment (+Pter). **a**. Tumor volumes were measured once every two days. Red arrows indicate the timed of pterostilbene administration. **b**. Tumor weights were calculated and are shown as a plot with the median and whiskers from minimum to maximum. **c**. The representative images of tumors in each group are shown. **d**. Paraffin-embedded tissue sections of the above tumors were subjected to H&E staining (X 40, left panels) and immunohistochemical (IHC) staining using the antibody against ER-α36 (X 100, right panels). (**p<0.01, NS: no significance).

The *in vitro* finding that silencing ER-α36 expression reduces the sensitivity of breast cancer cells to pterostilbene led to supposing that ER-α36 is a therapy target for pterostilbene. A xenograft mouse model was further used to study the effect on xenograft tumor growth in nude mice *in vivo*. Mb231/Si36 and Mb231 cell suspensions were injected into the left and right breast pad, respectively, of each mouse. These mice were randomized into two groups, vehicle control (Veh) and pterostilbene treatment (+Pter), when the tumor size reached approximately 50 mm^3^. The ER-α36-depleted Mb231/Si36 tumors in the left breast pad of the control group mice show significant reduction in growth compared with ER-α36-positive Mb231 tumors in the right breast pad (*p*<0.001, Fig. 4A (a)). The tumor weight significantly reduced in the left breast pad injected with ER-α36-depleted Mb231/Si36 cells (*p*<0.001, Fig. 4B). Pterostibene treatment was observed to significantly reduce the growth rate of the Mb231 tumors compared with physiological saline treatment (vehicle control group) (*p*<0.05, Fig. 4A(b)). The growth rate of Mb231/Si36 tumors did not significantly reduce after pterostilbene treatment compared to that of the vehicle control group (Fig. 4A(c)). The similar phenomena in tumor weight were also observed (Fig. 4B). Figure 4C shows the representative images of tumors in each group.

The tumors were dissected and processed for standard H&E staining and immunohistochemical staining of ER-α36 protein expression to investigate whether the reduction of tumor growth is associated with the downregulation of ER-α36 expression. A large area of necrosis was found in Mb231 xenograft tumors after pterostilbene treatment. However, the areas of necrosis in Mb231/Si36 tumors after pterostilbene treatment are small and scattered, which is similar to that of the vehicle (Fig. 4D). Pterostilbene was also found to inhibit ER-α36 expression to some extent in Mb231 xenograft tumors (Fig. 4D). The ER-α36 protein expression of Mb231/Si36 tumors with knockdown ER-α36 is negative (Fig. 4D). These results are consistent with previous *in vitro* studies, showing that silencing ER-α36 inhibits ER-negative breast cancer proliferation [24], [28]. The observations indicate that ER-α36 is a critical therapeutic target for breast cancer, and pterostilbene might be an ER-α36 inhibitor for ER-α36-positive breast cancer therapy.

## Discussion

Breast cancer is the most common malignant tumor, and is the major cause of cancer-related death in women in the world [30]. TAM treatment has substantially reduced the recurrence rates and mortality rates of breast cancer patients with ER-positive tumors [31]. However, the initial responsiveness to TAM therapy is limited because most advanced breast tumors recur with acquired resistance [5], [6]. Approximately 30% of breast cancers are ER-negative, and do not respond to endocrine therapies. Identifying a novel target is therefore an urgent topic of research. Previous studies report that ER-α36, a variant of conventional ER, is highly expressed in ER-negative tumors, and poorly expressed in ER-positive tumors [25], [32]. The acquired TAM resistance could nevertheless be mediated by ER-α36. If patients with ER-α66-positive breast cancer tumors have high ER-α36 expressions, they would be more resistant to TAM therapy than those with ER-α66-positive/ER-α36-nagetive tumors [21], [33]. The published literature suggests that ER-α36 is a critical therapeutic target for personalized therapies for ER-α66-negative/ER-α36-positive breast cancer and ER-α66-positive/ER-α36-high positive tumors with acquired TAM resistance. Finding agents which inhibit ER-α36 is therefore also our central issue. Pterostilbene is an antioxidant primarily found in blueberries, and is also considered to inhibit breast cancer, regardless of conventional estrogen receptor (ER-α66) status, by inducing apoptosis.

Two pairs of breast cancer cells, Mb231 and Mb231/Si36 cells and MCF-7 and MCF-7/ER36 cells, were used in this study. The ER status of Mb231 cells is ER-α66-negative/ER-α36-positive (Fig. 1A-C), therefore its ER-α36 was knocked down to investigate the role of ER-α36 in pterostilbene-induced antiproliferation on ER-α66-negative/ER-α36-positive breast cancer cells. ER-α36 knockdown was found to desensitize Mb231/Si36 cells to pterostilbene treatment (Fig. 1D). Pterostilbene treatment was also found to drastically reduce the *in vivo* proliferation capacity of Mb231 tumors with ER-α36-positive expression (Fig. 4A(b)). Clinical studies additionally demonstrated that approximately 40% of patients with ER-α66-positive tumors have high ER-α36 expression, and ER-α36 overexpression is associated with poor disease-free survival and disease-specific survival in patients with ER-α66-positive breast cancer [21]. Moreover, preclinical studies reveal that the pterostilbene-induced apoptosis in Mb231 is more obvious than in MCF-7 [14]. MCF-7/ER36 cells with ER-α36 overexpression were therefore used to investigate whether breast cancer cells with ER-α36 overexpression obtain more benefit from pterostilbene treatment. Overexpressing ER-α36 in MCF-7/ER36 cells increased the sensitivity of cells to pterostilbene (Fig. 1D) and enhanced pterostilbene-induced apoptosis (Fig. 2A and B), as expected.

The MAPK/ERK pathway is a major intracellular communication in breast cancer [34], and PI3K/Akt pathway is also very important in cell proliferation and survival by inhibiting apoptosis [35]. Pterostilbene has been revealed to inhibit PI3K/Akt activation in breast cancer to suppress the heregulin-β1/HER-2-modulated invasive and aggressive phenotype of breast cancer cells [16], [36], [37], and reduced matrix metalloproteinase 9 (MMP) expression, which is an enzyme implicated in micrometastasis [16]. Previous studies have additionally reported that the ERK1/2 tumorigenic pathway in cancer cells could also be inhibited by pterostilbene treatment [17], [36], [38]. Pterostilbene was found to inhibit ER-α36 expression in ER-α36-positive Mb231 cells and ER-α36-overexpressing MCF-7/ER36 cells (Fig. 2C), and ERK1/2 and Akt activation in Mb231 cells and MCF-7/ER36 cells in dose- and time-dependent manners, but not in parental MCF-7 cells. ERK1/2 and Akt phosphorylation were found to be abolished in Mb231/Si36 cells with ER-α36 knockdown (Fig. 3). ER-α36 may therefore be associated with MAPK/ERK and PI3K/Akt pathways, and ER-α36 knockdown directly inhibits ERK1/2 and Akt activation, which is consistent with previous studies which report that both MAPK/ERK and PI3K/Akt signaling activation are regulated by ER-α36 in breast cancer [19], [23], [25], [29], [39], [40]. Ohshiro *et al*. also demonstrated that estradiol and anti-estrogenic agent treatment activated ERK1/2 only with the presence of ER-α36, but not ER-α66 [29]. ER-α36 expression was also found to be decreased in tumor tissues after pterostilbene treatment (Fig. 4D). These studies suggested that the possible mechanism of the antiproliferative effect of pterostilbene in ER-α66 negative breast cancer might inhibit MAPK/ERK and PI3K/Akt signal pathways via ER-α36. Further investigations are needed to elucidate the exact mechanisms.

Pterostilbene treatment has been suggested to increase caspase 3 activity and expression, a critical mediator of mitochondrial apoptosis, and pterostilbene also could induce both caspase-dependent apoptosis and caspase-independent apoptosis in breast cancer cells [12], [14], [15]. In our study, caspase 3 expression was also found to be increased after pterostilbene treatment in ER-α36-positive Mb231 cells, but not in ER-α36-negative Mb231/Si36 cells (Fig. 2C). The pterostilbene-induced caspase-dependent apoptosis might be dependent on ER-α36. However, Mena *et al*. recently demonstrated that caspase-independent apoptosis is also induced by pterostilbene, the mechanism of which involves lysosomal membrane permeabilization, and caspase 3 activity failed to increase after pterostilbene treatment in MCF-7 cells [15]. The caspase 3 expression was consistently shown in this tudy to be unchanged under pterostilbene treatment in MCF-7 cells with or without ER-α36 overexpression (Fig. 2C). The pathways of pterostilben-induced-apoptosis in MCF-7 and MCF-7/ER36 cells are therefore different from that in Mb231. Possibly, the underlying mechanism may involve lysosomal membrane permeabilization [15]. This speculation must be further investigated and verified.

Pterostilbene failed to inhibit ER-α36 expression, ERK1/2 and Akt activation in ER-α66-positive MCF-7 cells, though their ER-α36 expression was positive. However, the inhibition could be restored by ER-α36 overexpression (Fig. 3), probably because ER-α36 overexpression competes with ER-α66 for DNA-binding elements in estrogen-responsive genes, inhibiting the estrogen-dependent and estrogen-independent transactivation activities of ER-α66 [19]. ER-α66 has also been previously reported to suppress ER-α36 promoter activity in an estrogen-independent manner, which could be released by ER-α36 [22]. The ER-α66 protein expression in MCF-7/ER36 cells decreased after transfection with ER-α36 overexpression compared with parental MCF-7 cells (Fig. 1C), which is consistent with a previous study [33]. The proliferation of MCF-7/ER36 cells might thus be dependent on ER-α36-activated MAPK/ERK and PI3K/Akt signaling, which could be blocked by pterostilbene treatment. This warrants future studies on the exact mechanisms of pterostilbene therapy against ER-α66-positive/ER-α36-positive breast cancer.

In summary, strong evidences for a key role of ER-α36 in pterostilbene treatment against ER-α66-negative/ER-α36-positive breast cancer cells both *in vitro* and *in vivo* are provided in this study. A possible role for ER-α36 in mediating pterostilbene sensitivity in ER-α66-positive/ER-α36-positive breast cancer cells is demonstrated *in vitro*. High ER-α36 expression is demonstrated for the first time to promote pterostilbene-induced apoptosis in breast cancer cells and necrosis in xenograft tumors. Furthermore, ER-α36 expression knockdown in Mb231/Si36 cells reduced the proliferation rate of tumors *in vivo*, together with decreased p-ERK1/2 and p-Akt expression. These finding suggested that ER-α36 is a therapeutic target in ER-α36-positive breast cancer tumors which have resistance to TAM. Pterostilbene is a selective inhibitor that targets ER-α36 in the future therapy against ER-α36-positive breast cancer.
